# Modified Hughes procedure for reconstruction of large full-thickness lower eyelid defects following tumor resection

**DOI:** 10.1186/s40001-016-0221-1

**Published:** 2016-06-30

**Authors:** Ahmed M. Hishmi, Konrad R. Koch, Mario Matthaei, Edwin Bölke, Claus Cursiefen, Ludwig M. Heindl

**Affiliations:** Department of Ophthalmology, University of Cologne, Cologne, Germany; Department of Radiology and Radiooncology, University of Duesseldorf, Duesseldorf, Germany

**Keywords:** Hughes flap, Tarsoconjunctival flap, Modified Hughes procedure, Oculoplastic surgery, Lower eyelid tumor

## Abstract

**Background:**

Tarsoconjunctival flap advancement, or the Hughes procedure, is among the techniques of choice for reconstructing full-thickness lower eyelid defects so as to restore normal anatomy and function with the best possible cosmetic outcome. The purpose of this study is to report the outcome of a series of patients treated with a modified Hughes procedure following malignant tumor removal.

**Methods:**

This retrospective study included 45 consecutive cases of modified Hughes procedures performed between January 2013 and October 2015. During Hughes flap creation an incisional plane was chosen in all cases, which left Müller’s muscle attached to the superior tarsal margin, while disinserting the levator aponeurosis. All cases were grouped according to the horizontal length of the lower lid defect to be reconstructed, as well as to the type of anterior lamella reconstruction (free graft vs. inferiorly based advancement flap). Grouped data were compared for the rate of surgical success, defined as achievement of normal lid function and satisfactory cosmesis without needing further surgical interventions, and for the frequency of specific complications.

**Results:**

Surgical success was achieved in 39 cases (87 %). The remaining cases required additional surgery for minor complications including lower-lid ectropion (4 %), pyogenic granuloma (4 %), or lower lid margin hypertrophy (2 %). Donor-site complications were not detected apart from one case of mild entropion with focal trichiasis. No case of premature flap rupture was seen. Neither the horizontal length of the lower lid defect (*p* = 0.489), nor the type of anterior lamella reconstruction (*p* = 0.349) significantly affected the surgical success. Particularly, there was no increased onset of lower-lid ectropion among patients receiving an advancement flap.

**Conclusions:**

The modified Hughes procedure remains a well-suited technique for reconstructing lower eyelid defects involving up to 100 % of the horizontal lid length. Leaving Müller’s muscle attached to the Hughes flap might prevent premature flap dehiscence without increasing the frequency of upper lid retractions in turn. Whether using a free skin graft or a skin-muscle advancement flap for anterior lamella reconstruction, seems to be insignificant for the functional-aesthetical outcome.

## Background

Depending on the involvement of the horizontal eyelid margin, several techniques have been used for the reconstruction of full-thickness eyelid defects [1–4]. These include Tenzel semicircular rotation flap, free tarsoconjunctival graft and Mustarde cheek rotation flap and the one being studied here, the tarsoconjunctival flap advancement (Hughes procedure). The latter technique, even though a similar approach had been described already in 1911 by Koellner [5], was popularized—and thus the name—by Dr. Wendel L. Hughes, (Bellevue Hospital, New York) [6]. He first presented his technique in 1937 [7], a few years before publishing his ground-breaking comprehensive thesis “Reconstructive Surgery of the Eyelids”, which at that time was without equal in the field of oculoplastic surgery books [6, 8].

Using this approach Dr. Hughes followed the widely acknowledged principal introduced by Gradenigo, that reconstruction of lid structures is best and most satisfactorily achieved using healthy tissue of lid origin as well, thus to replace “like with like” [9]. The procedure itself is a two-stage, eyelid-sharing technique for the reconstruction of full-thickness defects, which comprise at least 50 % (and up to 100 %) of the horizontal lower eyelid margin. The first stage involves (a) the advancement of a tarsoconjunctival flap, the “Hughes” flap, from the upper to the lower eyelid to reconstruct the posterior lamella, consisting of tarsus and conjunctiva, and (b) the reconstruction of the anterior musculocutaneous lamella either using a free full-thickness skin graft, or a local skin, or skin-muscle advancement flap. After several weeks, which allow new vasculature to form within and around the anterior and posterior grafts, and during which the affected eye remains covered by the blood-supplying flap pedicle, the latter is finally divided at the level of the new lower lid margin in a second stage procedure. Due to the temporary eyelid closure Hughes procedures are not the treatment of choice in one-eyed patients, who need eyelid reconstruction on the side of their only seeing eye.

Over the decades the procedure has further evolved. Major modifications introduced by Hughes himself and others included the sparing of the marginal upper lid tarsus and the removal of the levator muscle complex from the tarsoconjunctival flap, which especially reduced the frequency of donor-site, i.e. upper lid complications, such as upper lid retraction, entropion, and trichiasis [10, 11].

In this modified form the Hughes procedure is widely used to date as the technique of choice for the reconstruction of substantial horizontal full-thickness defects of the lower eyelid given the low rate of complications, the superior functional and esthetical outcome and high patient satisfaction. In this study, we evaluate our experience with the modified Hughes procedure with regard to the functional and esthetical outcome and typical complications.

## Methods

### Patients

This study included consecutive patients who underwent a modified Hughes procedure for lower eyelid reconstruction at the Department of Ophthalmology, University of Cologne, from January 2013 to October 2015. The need for ethical approval was waived by the ethical committee of the University of Cologne because of the retrospective nature of this work (#16-164). Inclusion criteria for patients were (a) histopathologically confirmed R0 resection of malignant skin tumors of the lower eyelid, (b) full-thickness lower eyelid defects comprising conjunctiva, tarsus (complete vertical lack), orbicularis and skin, of at least 50 % up to 100 % of the horizontal lid fissure length, leaving a residual fibro-tarsal stump both temporally and nasally for Hughes flap attachment, (c) one surgeon (LMH), (d) standardized technique of the Hughes procedure as specified below, and (e) a follow-up time of at least 1 month after separation of the tarsoconjunctival Hughes flap. Patients, in which a several week lasting lid closure would have been contraindicated, such as children at risk of occlusion amblyopia and monocular patients, underwent different reconstructive measures and were not included.

For all included patients the underlying oncological causes were assessed, as well as the size and the position of eyelid defects. Lower eyelid defects between 50 and 59 % of the horizontal (canthus to canthus) lid length were graded as “small”, between 60 and 79 % as “medium”, and defects of ≥80 % as “large”.

### Description of the standardized modified Hughes procedure

**Fig. 1:**
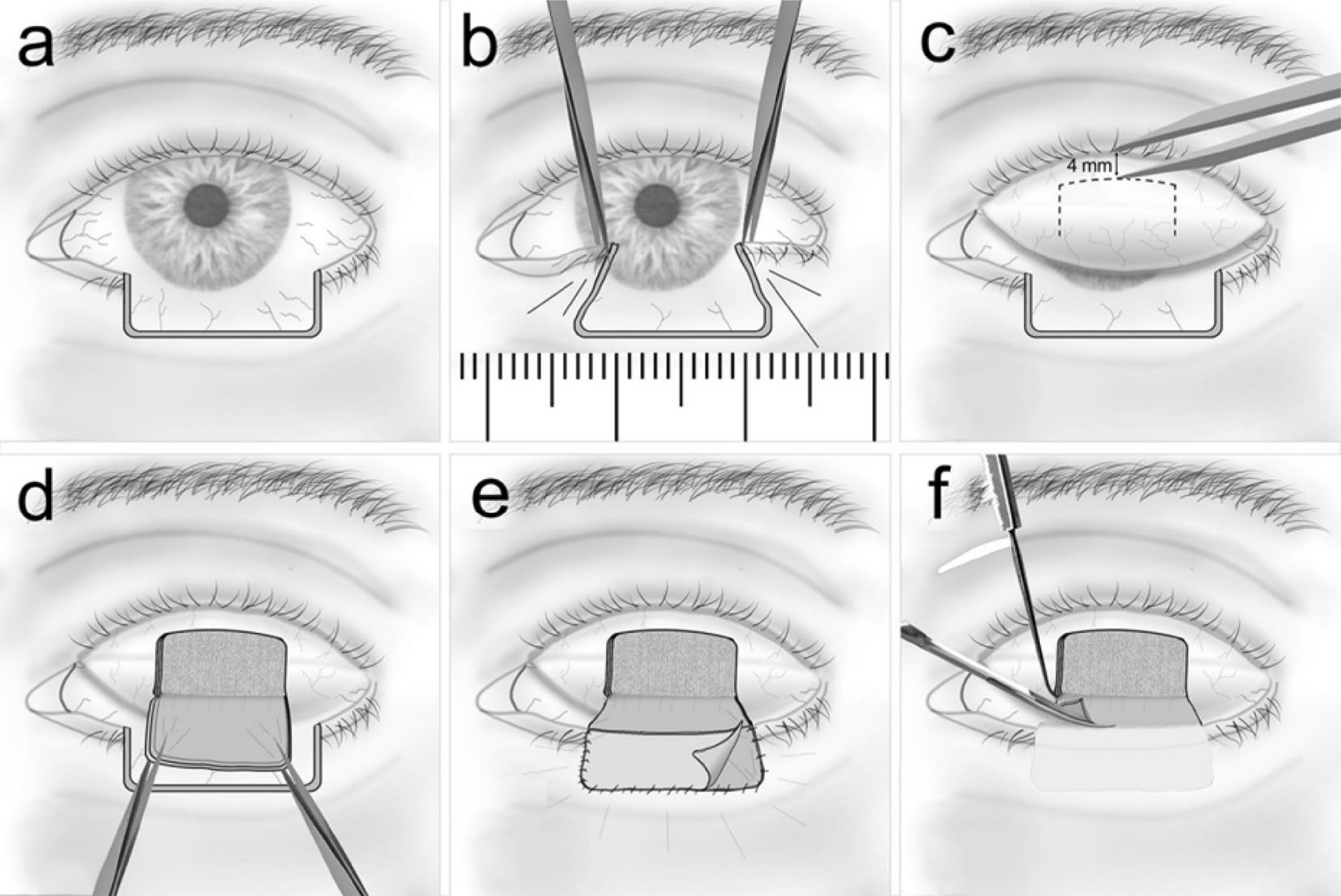
Diagram illustrating the basic steps of the modified Hughes procedure. **a** *Left* eye with a full-thickness lower eyelid defect involving >50 % of the *horizontal* lid length. **b** Approximation of the temporal and nasal wound margins using two pairs of forceps to measure the required width of the Hughes flap. **c** Everting the upper lid to expose the conjunctiva and measuring 4 mm of the marginal tarsus to be preserved. **d** Tarsoconjunctival flap is cut and extended down to cover the defected area. **e** Suture fixation of the Hughes flap (posterior lamella) and of a free skin graft (anterior lamella), which has been harvested from the contralateral upper eyelid. **f** Division of the pedicle about 0.5 mm above the lower lid margin, performed 6 weeks after Hughes flap fixation

In all patients, local anesthetic solution consisting of a mixture of xylocaine 1.0 % and adrenaline (epinephrine) 1:200,000 unbuffered was injected around the lower eyelid defect, and into the upper eyelid subcutaneously and subconjunctivally before dissection of the Hughes tarsoconjunctival flap. Prior to this, excisional surgery of malignant eyelid tumors with overnight histopathological processing and evaluation had been performed, repeatedly if required, until tumor-free resection boundaries of the full thickness lower eyelid defect, i.e. a pR0 resection, were confirmed. Pulling the medial and lateral boundaries centrally with two pairs of forceps and measuring the resulting distance with a millimeter scale determined the required tarsoconjunctival flap length (Fig. 1b).

**Fig. 2:**
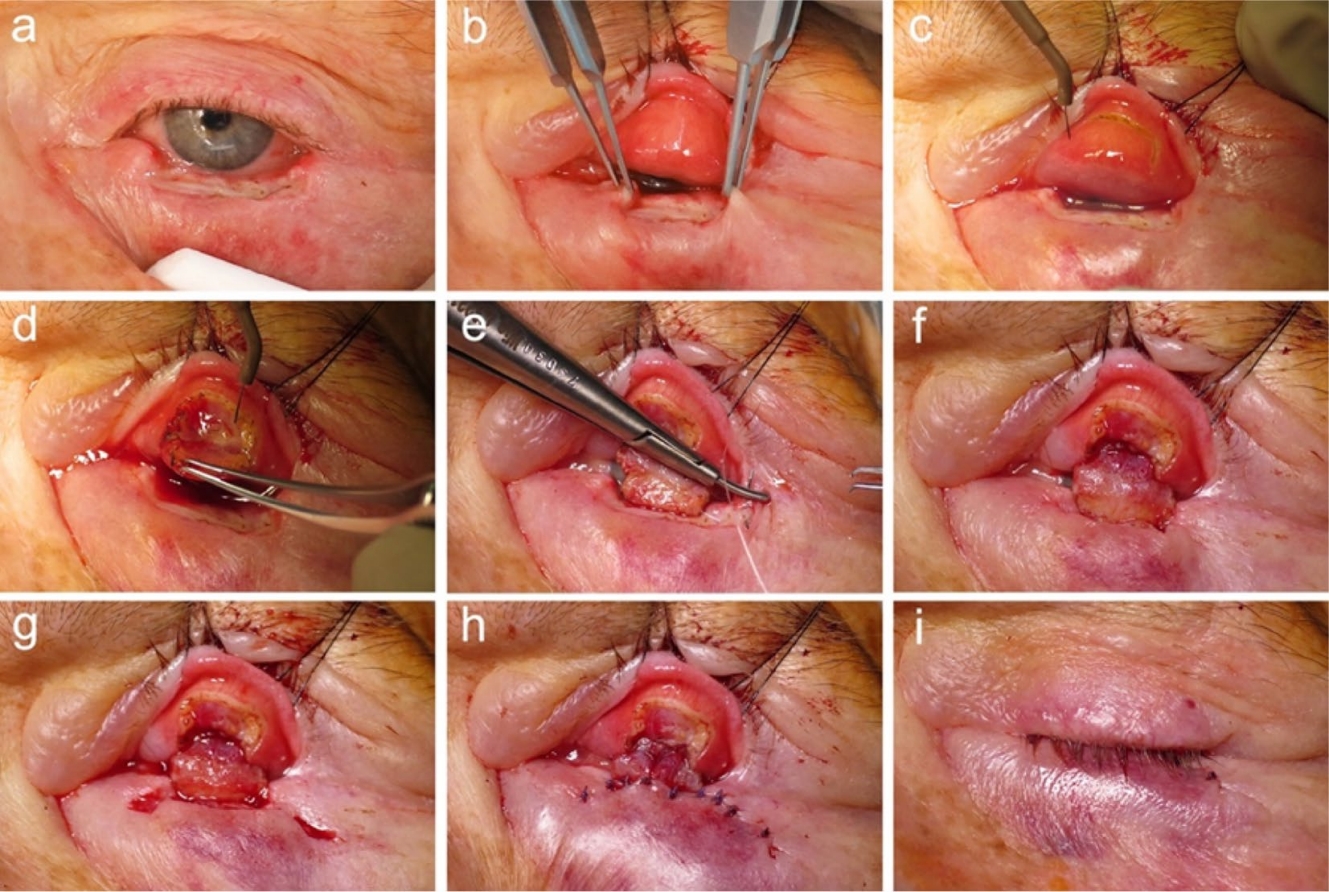
Photographs of a modified Hughes procedure. **a** A patient with a full-thickness lower eyelid defect with histopathologically confirmed tumor-free boundaries after BCC excision. **b** Measuring the defect size and the subsequently required Hughes flap width. **c** Incision in an inverted U shaped manner through conjunctiva and the full thickness of the tarsus. **d** Dissection of all fibromuscular levator aponeurosis attachments from the anterior tarsal surface **e**, **f** Edges of the Hughes flap are sutured to the remnants of the medial and lateral tarsus of the lower eyelid. **g** Preparation of an inferiorly based skin-muscle advancement flap. **h** Fixation of the advancement flap with absorbable sutures to the lateral wound margins and posteriorly to the Hughes flap. **i** The *left eye* post-surgically (before the pedicle division)

The upper eyelid was everted using a Desmarres retractor. Markings were done on the everted conjunctiva in such a manner that the distal incision on the conjunctival side of the upper lid was 4 mm away from the lid margin (Fig. 1c). Incision through the full-thickness of the tarsus was made in an inverted U shaped manner over the upper lid conjunctiva (Fig. 2c). The flap was raised by dissecting all levator aponeurosis attachments in the avascular pretarsal plane until the upper tarsal border was reached (Fig. 2d) leaving only attached the majority of inserting superior tarsal muscle (Müller’s muscle) fibers and the conjunctiva. Dissection in this plane between Müller’s and levator muscle was continued posteriorly until the flap could comfortably be advanced into the lower lid defect without any significant tension to the posterior lamella of the upper lid (Fig. 1d). The flap was sutured tarsus to tarsus into the defect using non-absorbable suturamid 6.0 sutures (Fig. 2e, f), thereby recreating the posterior lamella of the lower lid.

Hereafter, for recreation of the anterior lamella, either a free skin graft was inset from the ipsi- or contralateral upper lid (Fig. 1e), or an inferiorly based skin-muscle flap was advanced upwards (Fig. 2g). The final choice between these two options was left to the patient’s discretion after detailed explanation, also taking into account whether sufficient excess skin was available at the ipsi- or the contralateral upper eyelid. The skin was sutured using absorbable Vicryl 6.0 stitches (Figs. 1e, 2h). Chloramphenicol ophthalmic ointment and a bulky dressing were applied.

According to the procedural standard of our institution flap division was undertaken 6–8 weeks after the Hughes procedure under local anesthesia to minimize the risk of flap ischemia and necrosis, although according to newer studies a much shorter interval of 2 weeks or even less might be sufficient for flap revascularization [12, 13]. By everting the upper lid, the conjunctival and Müller muscle pedicle flap was cut 0.5 mm above the area of the skin inset allowing the mucocutanous line to heal by secondary intention (Fig. 1f). If necessary, the superior edge of the skin and conjunctiva was revised to remove granulation tissue from the new lower lid margin.

**Fig. 3:**
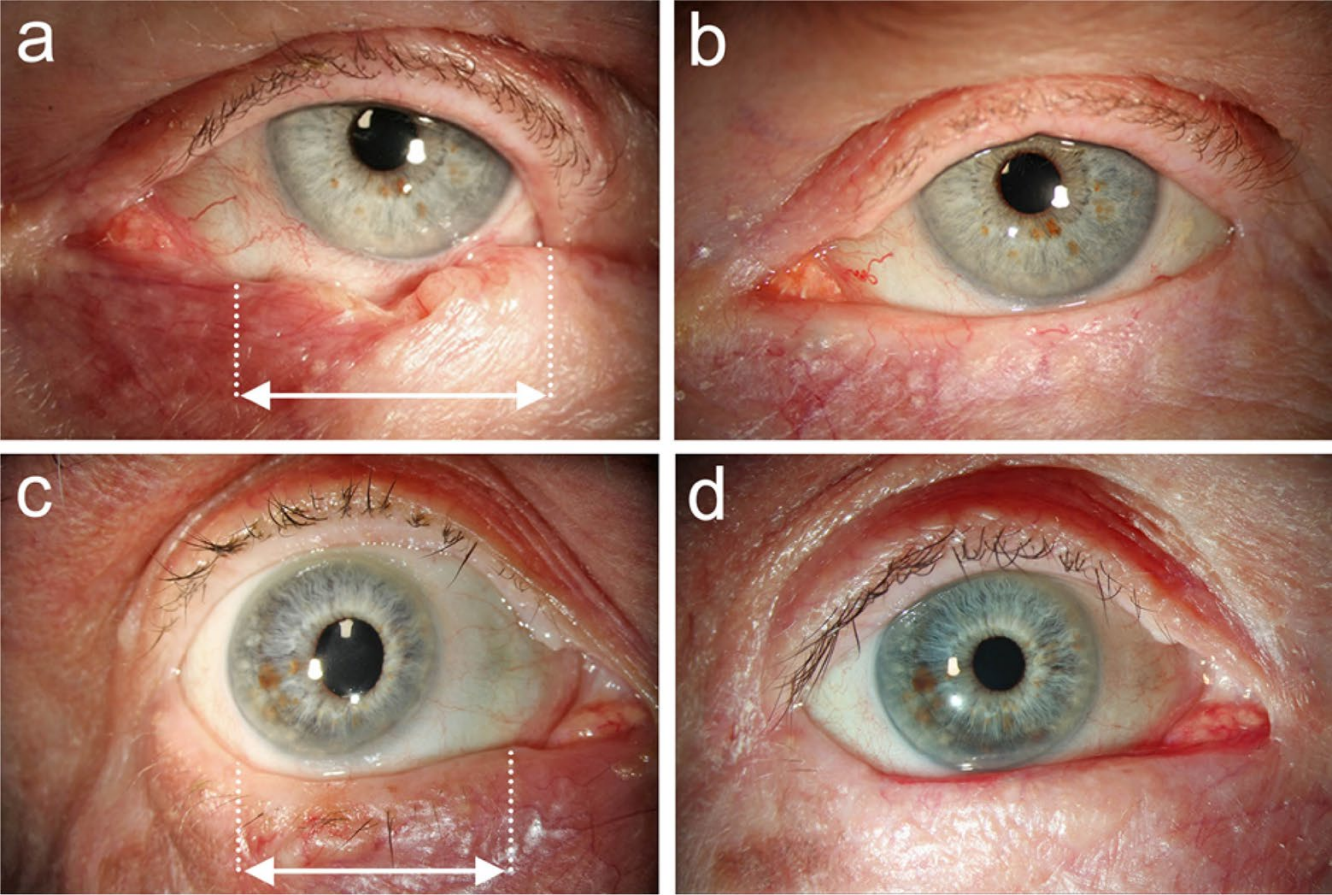
Clinical images of two cases, both before malignant tumor excision and several months after Hughes flap division. **a** A 75-year-old patient with an ulcerative lower lid tumor in the *left eye* histopathologically proving to be a basal cell carcinoma (*arrow*: horizontal extent of the lid defect following pR0 resection). **b** Full recovery with normal lid function, normal lid position, and satisfactory cosmesis 30 months after Hughes flap division. **c** A 77-year-old male patient with a nodular lower lid tumor and focal eyelash loss in the *right eye*. Histopathological evaluation revealed a basal cell carcinoma. **d** Normal lower lid function and good esthetical outcome 10 months after separation of the Hughes flap

The data of all included patients were analyzed for the frequency of known postsurgical complications of Hughes procedures. The latter were grouped into “early complications” arising between tarsoconjunctival flap advancement (stage 1) and flap separation (stage 2), such as premature flap dehiscence, flap necrosis, and into “late complications” occurring after flap separation. The latter group was further stratified into “donor site complications”, including upper lid retraction and trichiasis, and “lower lid complications”, including lower lid ectropion, entropion, trichiasis, lid margin irregularities. Surgical success of the modified Hughes procedure was constituted when a normal lid function and a satisfactory cosmesis were achieved without the need for additional surgical measures. Satisfactory cosmesis was judged based on the patients’ satisfaction as documented in the patient record at the last follow-up visit, and also by the physicians based on standardized follow-up photographs, that were taken from each patient (Fig. 3b, d) 3 months after flap separation and repeatedly after.

Patients were grouped (a) depending on the technique chosen for anterior lamella reconstruction (free skin graft vs. skin-muscle advancement flap) and (b) depending on the horizontal defect length, and grouped data were analyzed for surgical success rates and frequencies of complications. Pearson’s Chi square test was chosen for statistical analyses, which were performed using SPSS (v. 21.0, IBM, Chicago, IL, USA) software. A *p* value <0.05 was considered statistically significant.

## Results

Between January 2013 to October 2015, forty-four (22 female) consecutive patients underwent 45 modified Hughes procedure after malignant eyelid tumor resection. In one female patient eyelid malignancies developed first in the left, later in the right lower eyelid, thus requiring reconstructive surgery in both eyes. The mean age of the patients was 75 years (SD 11.6, range 40–96). The right eye was affected in 21 cases (47 %).

The underlying histopathologically confirmed lower eyelid malignancies included 36 cases (80 %) of basal cell carcinoma (BCC), 7 cases (16 %) of squamous cell carcinoma (SCC), and 2 cases of other types (1 sweat gland adenocarcinoma; 1 conjunctival mucoepidermoid carcinoma).

Regarding the horizontal eyelid defect size, 17 cases (38 %) showed a small defect, 17 cases (38 %) showed a medium defect, and 11 cases (24 %) showed a large defect. The anterior lamella was reconstructed with a free skin graft in 22 cases (49 %), or with an inferiorly based skin-muscle advancement flap in 23 cases (51 %), respectively.

After flap division the mean follow-up time of the patients was 548 days (SD 312; range 32–1042), corresponding to 18 months.

Surgical success, defined as normal lid function and satisfactory cosmesis without the need for additional surgical measures (before or after Hughes flap dissection), was achieved in 39 cases (87 %), with two of those being depicted in Fig. 3.

In all remaining six cases a good functional and cosmetical result was finally achieved after additional surgical measures, which are specified below. The frequencies of all postsurgical complications observed in our patients stratified by the type of anterior lamella reconstruction are shown in Table 1.

**Table 1:** Frequencies of complications following modified Hughes procedures stratified by the type of anterior lamella reconstruction

Type of complications	All cases (*n* = 45)			Free skin grafts (*n* = 22)			Advancement flaps (*n* = 23)		
Early complications (before flap division)	1 (2 %)			–			1 (4 %)		
Flap suture dehiscence			1 (2 %)			–			1 (4 %)
Flap pedicle rupture			–			–			–
Flap necrosis			–			–			–
Late complications (after flap division)	6 (13 %)			5 (23 %)			1 (4 %)		
Donor-site complications (upper eyelid)		1 (2 %)			1 (5 %)			–	
Lid retraction			–			–			–
Entropion/trichiasis			1 (2 %)			1 (5 %)			–
Lower lid complications		5 (11 %)			4 (18 %)			1 (4 %)	
Ectropion			2 (4 %)			2 (9 %)			–
Trichiasis			–			–			–
Lid margin hypertrophy			1 (2 %)			1 (5 %)			–
Pyogenic granuloma			2 (4 %)			1 (5 %)			1 (4 %)
Tumor recurrence (lower eyelid)	2 (4 %)			1 (5 %)			1 (4 %)		

With regard to early complications during the 6-week time frame before the scheduled flap separation, we only observed altogether one single case (2 %) of suture dehiscence, which required re-suturing of the anterior and posterior lamellae on day 13 after the modified Hughes procedure. No further early complications were seen. Particularly, there was neither a case of flap pedicle rupture, i.e. of premature flap dehiscence, nor a case of flap necrosis.

After flap separation, lower lid complications observed in this study included two cases of ectropia that were diagnosed 11 days and 40 days after flap division. Corrective surgery by means of a lateral tarsal strip fixation was performed 4 months later. Two patients developed a pyogenic granuloma at the conjunctival flap wound interface, subsequently treated by granuloma excision. One case showed a persisting hypertrophy of the lower lid margin after flap separation caused by a protruding musculocutaneous pedicle stump. The lid margin was surgically corrected and straightened by excision of the protruding tissue.

The only donor-site complication occuring in this study was a moderate case of upper lid entropium with nasally accentuated trichiasis, which was satisfactorily corrected via cryoepilation of the respective upper lid eyelashes. Meanwhile, not a single case of upper eyelid retraction was observed.

Two patients, which had been treated for lower eyelid BCC, developed lower eyelid tumor recurrences and thus required further multistep treatment with tumor resection and lower lid re-reconstruction. For the evaluation in this study, these two cases were nevertheless categorized as surgically successful, since such tumor recurrences are not attributable to the surgical technique of the modified Hughes procedure itself but in fact to a microscopically incomplete tumor resection prior to any reconstructive measure.

The horizontal length of the lower eyelid defect to be reconstructed did neither significantly affect the surgical success rate (*p* = 0.489), nor the frequency of specific Hughes procedure related complications.

No significant difference in the surgical success rate was detectable between patients receiving a free skin graft and those treated with an advancement flap for anterior lamella reconstruction (*p* = 0.349). Equally, no differences were observed with regard to specific complications. In particular, the number of lower lid ectropia after Hughes flap dissection did not significantly differ between both groups (*p* = 0.139). In the only two ectropion cases we observed, the anterior lamella had been reconstructed using a free skin graft.

## Discussion

The high functional-esthetical success rate of the modified Hughes procedure, observed in this study, corresponds with the beneficial results, which have been reported in previous publications [14–17]. Also, with regard to the vast majority of BCC followed by SCC as underlying tumor entities, the study population shows a typical distribution [14, 18].

**Fig. 4:**
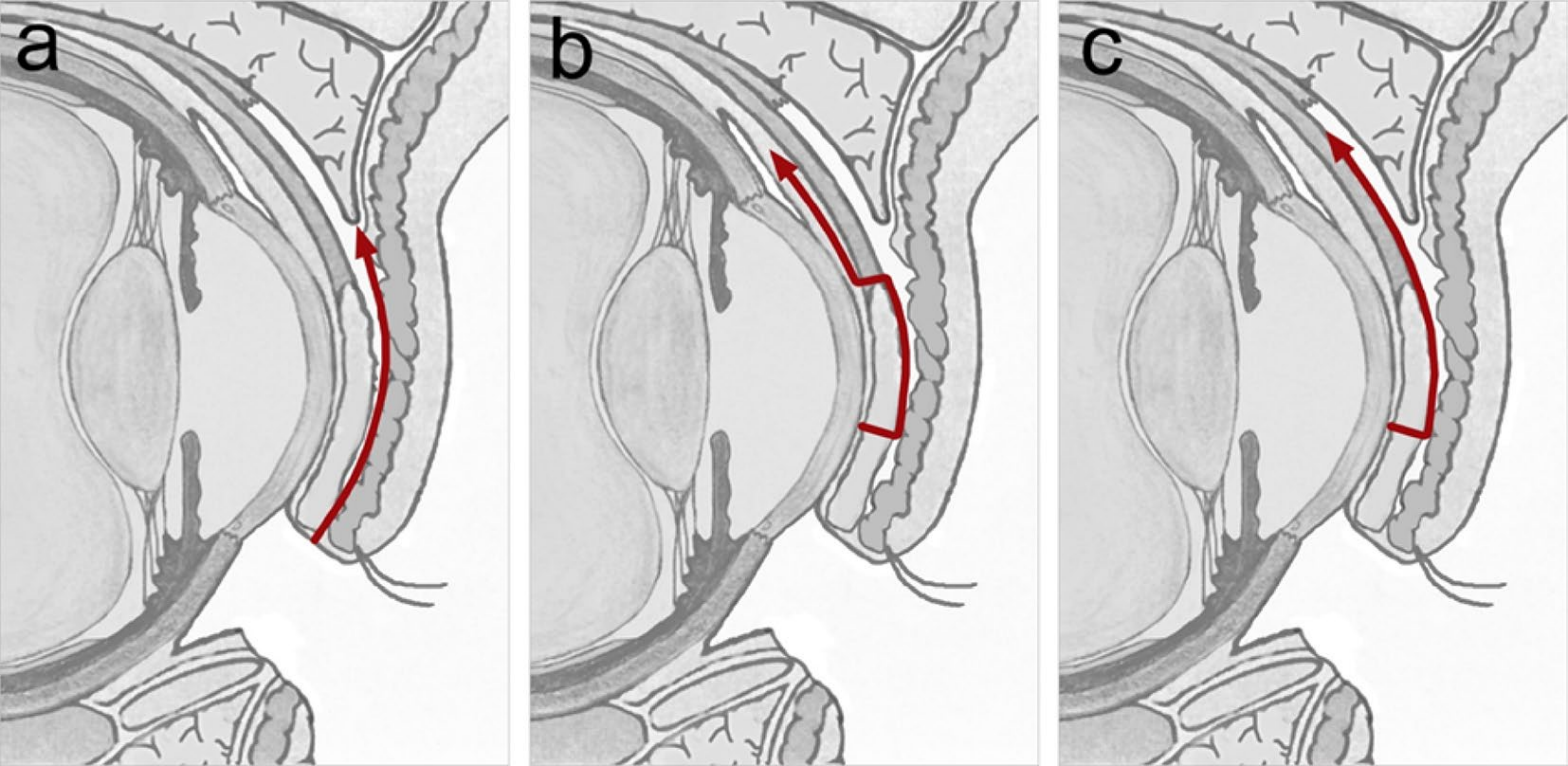
Incisional planes for harvesting a Hughes tarsoconjunctival flap. **a**. Incision of the classical Hughes procedure (*arrow*) starting at the *grey line* of the lid margin, leaving the levator muscle aponeurosis and Müller’s muscle attached to the tarsal plate. **b** Currently, the most widely used incisional plane spares 4 mm of the marginal tarsus. Levator and Müller’s muscle attachments are completely separated from the tarsus leaving only a thin solely conjunctival pedicle. **c** Incisional plane used in the present study. While disinserting the levator aponeurosis from the tarsus, Müller’s muscle insertions are left attached to the superior tarsal border

Altogether, we observed a very low rate of early and late complications. In particular, complications at the Hughes graft donor-site comprising namely upper lid retraction or upper lid entropium with trichiasis did only occur in one single case. Donor-site complications have more frequently been described in patients undergoing the classical Hughes procedure [19, 20], which has been attributed to differences in the incisional plane during flap preparation. Instead of sparing the marginal upper eyelid tarsus, the incision of the classical procedure starts at the lid marginal grey line and splits the upper eyelid over the entire tarsal height in a posterior and anterior lamella (Fig. 4a) [20], which has been shown to potentially result in upper lid instability causing entropium and trichiasis, as well as damage to the eyelash root bulbs. Furthermore, the dissection plane of the classical procedure between anterior and posterior lamella leaves the levator and Müller’s muscle complex attached to the tarsus (Fig. 4a). This has been hold responsible for frequently observed upper eyelid retractions, since, during the period of lid closure, the levator complex adheres in a stretched state to the anterior upper lid lamella. And once relieved after flap separation, it may contract again pulling the entire upper lid up- or rather backwards.

Over the decades, those donor-site complications lead to significant modifications resulting in a modified Hughes procedure [20]. Currently, most authors recommend to completely separate the levator as well as the Müller’s muscle from the superior tarsal border during Hughes flap preparation thereby leaving a very delicate and thin pedicle only consisting of conjunctiva for tarsal blood supply (Fig. 4b) [14]. In this study a different incisional plane—previously described by McCord and Nunery—was chosen [21], which indeed separates the entire levator muscle from the tarsus, while leaving the majority of Müller’s muscle fibers attached to the superior tarsal margin (Fig. 4c). Thus, the flap pedicle remains thicker and, from our perspective, more robust. Notably, although Müller’s muscle remained attached to the tarsus, we did not observe a single case of upper lid retraction. The only donor-site complication occurring in this study was a single case of moderate entropium with trichiasis, a finding, which has been described as well in individual cases after complete Müller’s muscle disinsertion from the tarsus [14, 17]. In view of this fact, this surgical approach appears suitable not least because a more robust and thicker flap pedicle should reduce the risk of a premature flap dehiscence and consecutively of hypoperfusion and necrosis of the tarsal autograft. After all, such premature flap dehiscences have been reported in 8 % of cases, in which the flap pedicle consisted of a thin conjunctival layer only [22].

In around half of the cases presented here, the anterior lamella was reconstructed using a free skin graft from the upper eyelid, while a skin-muscle advancement flap was used in the remaining cases. We did not observe statistically significant differences in the surgical success rate of both groups. In particular, there was also no significant difference in the onset of lower eyelid ectropion in both groups, although some authors have attributed an increased risk of lower lid ectropion to using a local advancement flap for anterior lamella reconstruction [4, 23]. However, to our knowledge a statistical comparison of both reconstructive techniques in the context of Hughes procedures has not been performed in consecutive patients to date. In this study, the two single cases of lower eyelid ectropion even developed in patients undergoing free skin grafting. Those cases were thus more likely due to horizontal lower lid laxity caused by a horizontally oversized Hughes flap than due to a gravitation pull of the anterior lamella. According to our data, we would consider both techniques, free skin grafts and local advancement flaps, equally suited for anterior lamella reconstruction during Hughes procedures.

The shortcomings of this study are based on its retrospective nature. Also, with regard to the incisional plane of the Hughes flap, a control group is lacking. A prospective study should therefore be initiated to compare the functional-esthetical outcome, first depending on whether a solely conjunctival (Fig. 4b) or a musculoconjunctival flap pedicle (Fig. 4c) is created, and second depending on the chosen technique of anterior lamella reconstruction.

## Conclusions

This retrospective study confirms that the modified Hughes procedure remains a well-suited technique for repairing large full-thickness lower eyelid defects involving up to 100 % of the horizontal lid length. In around 90 % of cases it results in normal lid function and satisfactory cosmesis without any demand for further surgery.

By leaving Müller’s muscle attached to the superior tarsal border while harvesting the Hughes flap, a robust musculoconjunctival pedicle is formed, which might be less prone to premature flap dehiscence than a delicate solely conjunctival flap pedicle, without seeming to increase the frequency of upper eyelid retractions in turn.

For the functional-esthetical outcome of the modified Hughes procedure it finally seems to be insignificant whether a free skin graft or a skin-muscle advancement flap is used for reconstructing the anterior lamella of the lower eyelid.
